# Supplementary figures and images for: Probing the Structural Dynamics of the Unbound MAX Protein: Insights from Well-Tempered Metadynamics
Source: J Chem Inf Model. 2025 Dec 28;66(1):425–36. doi: 10.1021/acs.jcim.5c02155 (PMC12801308; doi:10.1021/acs.jcim.5c02155)

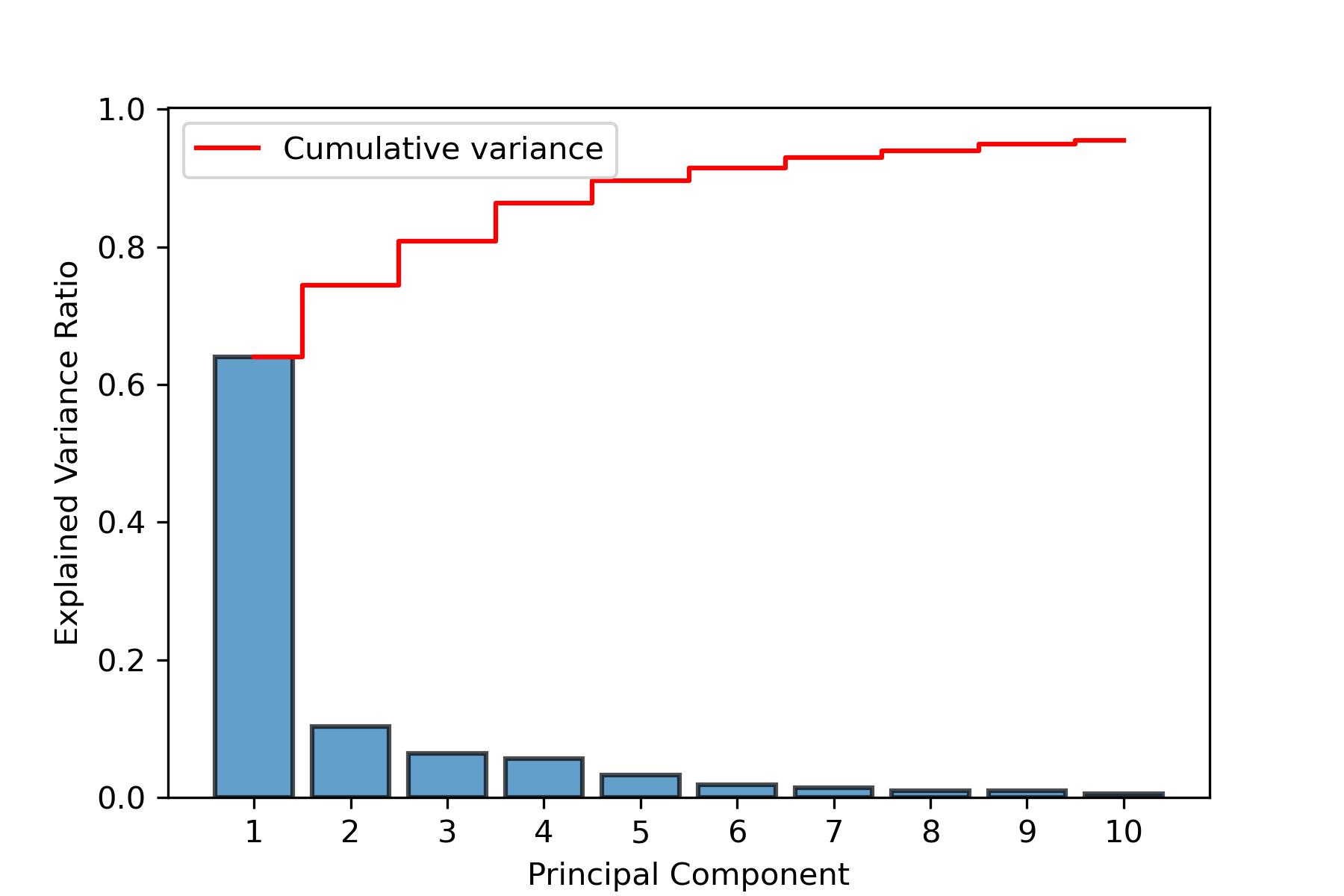

Supplement: Supplementary file 1 [file ci5c02155_si_001.zip › Supporting_infomation/SI-fig1.jpg]

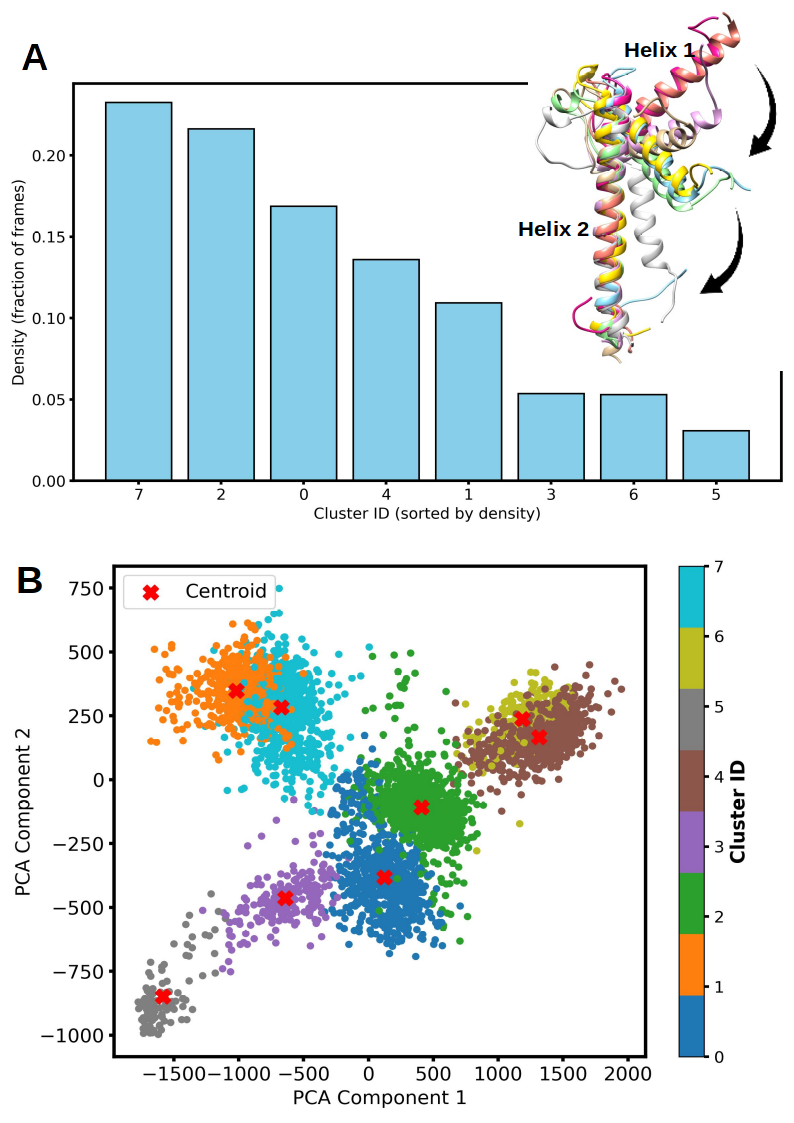

Supplement: Supplementary file 1 [file ci5c02155_si_001.zip › Supporting_infomation/SI-fig2.png]

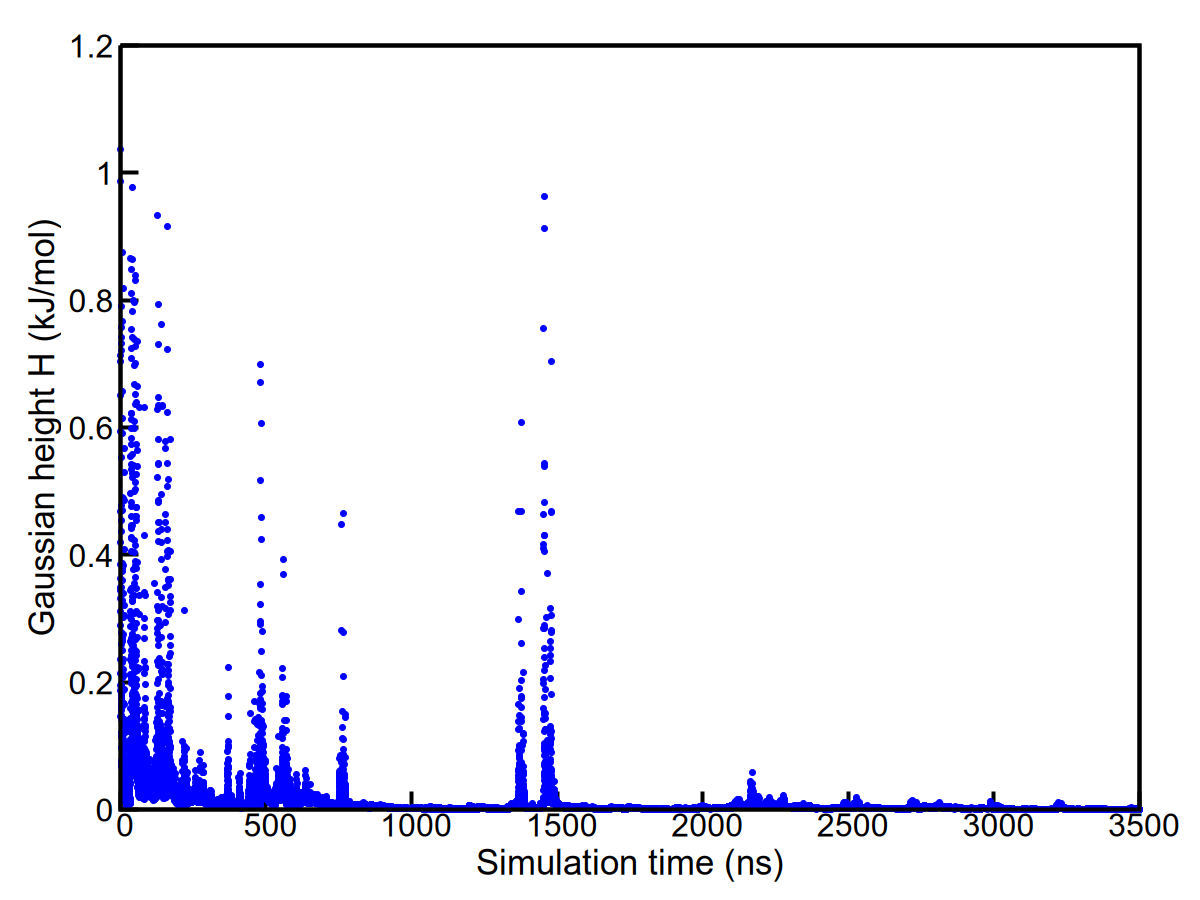

Supplement: Supplementary file 1 [file ci5c02155_si_001.zip › Supporting_infomation/SI-fig3.png]

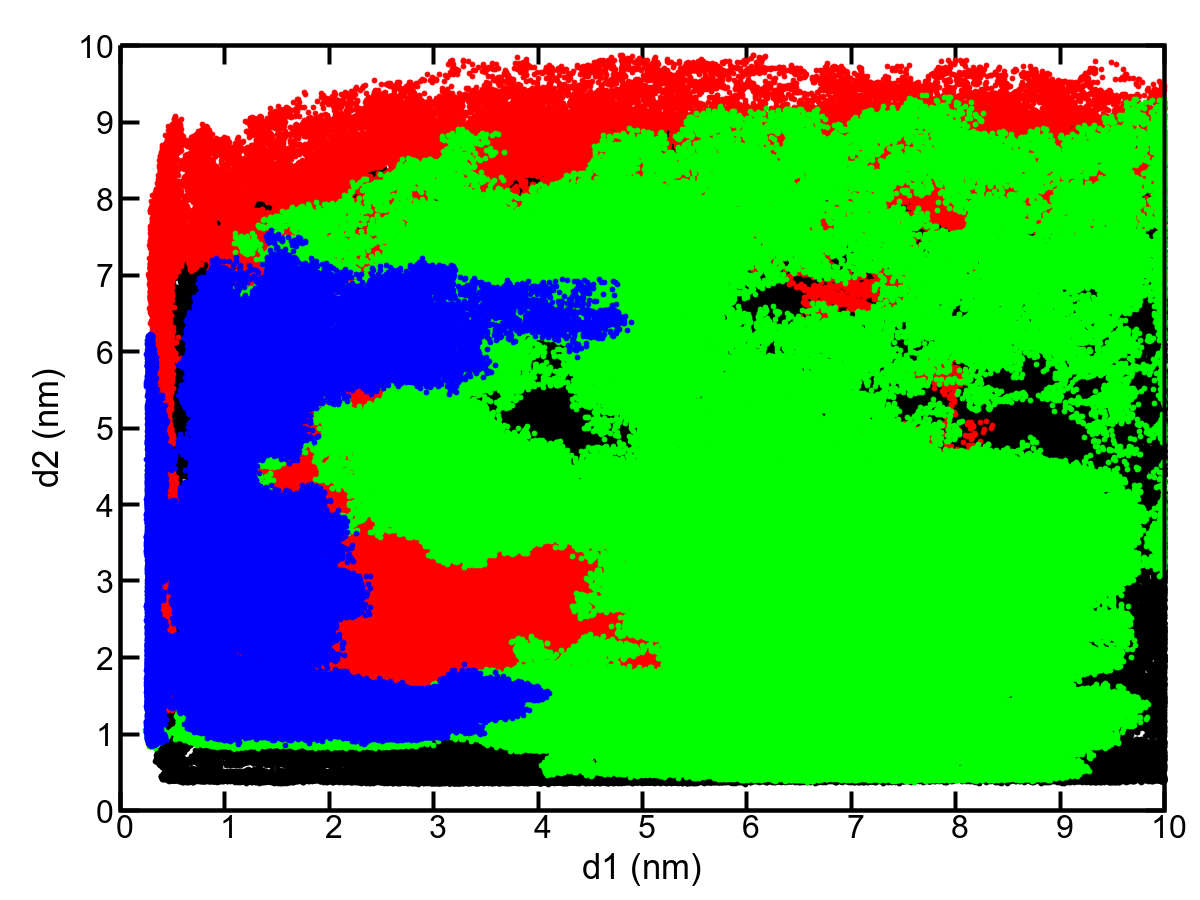

Supplement: Supplementary file 1 [file ci5c02155_si_001.zip › Supporting_infomation/SI-fig4.png]

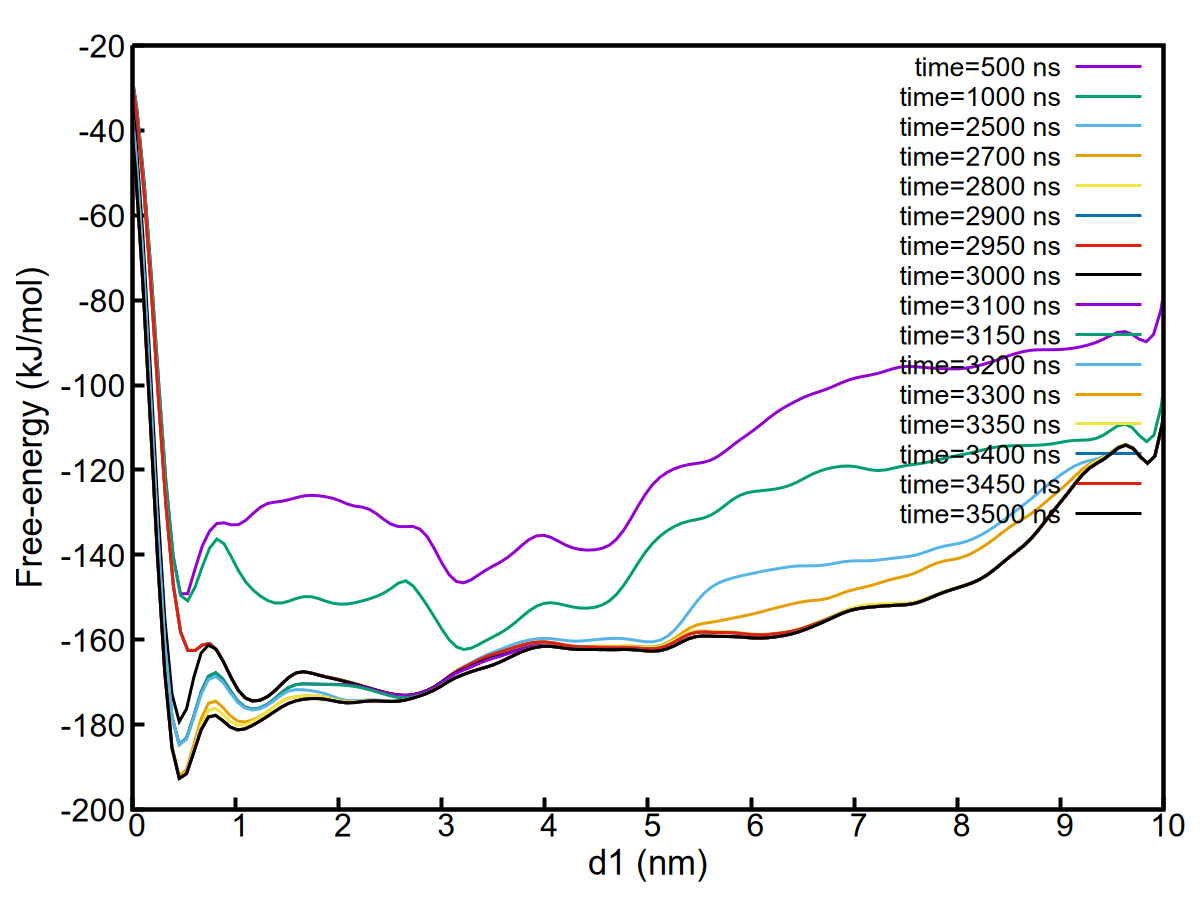

Supplement: Supplementary file 1 [file ci5c02155_si_001.zip › Supporting_infomation/SI-fig5-a.png]

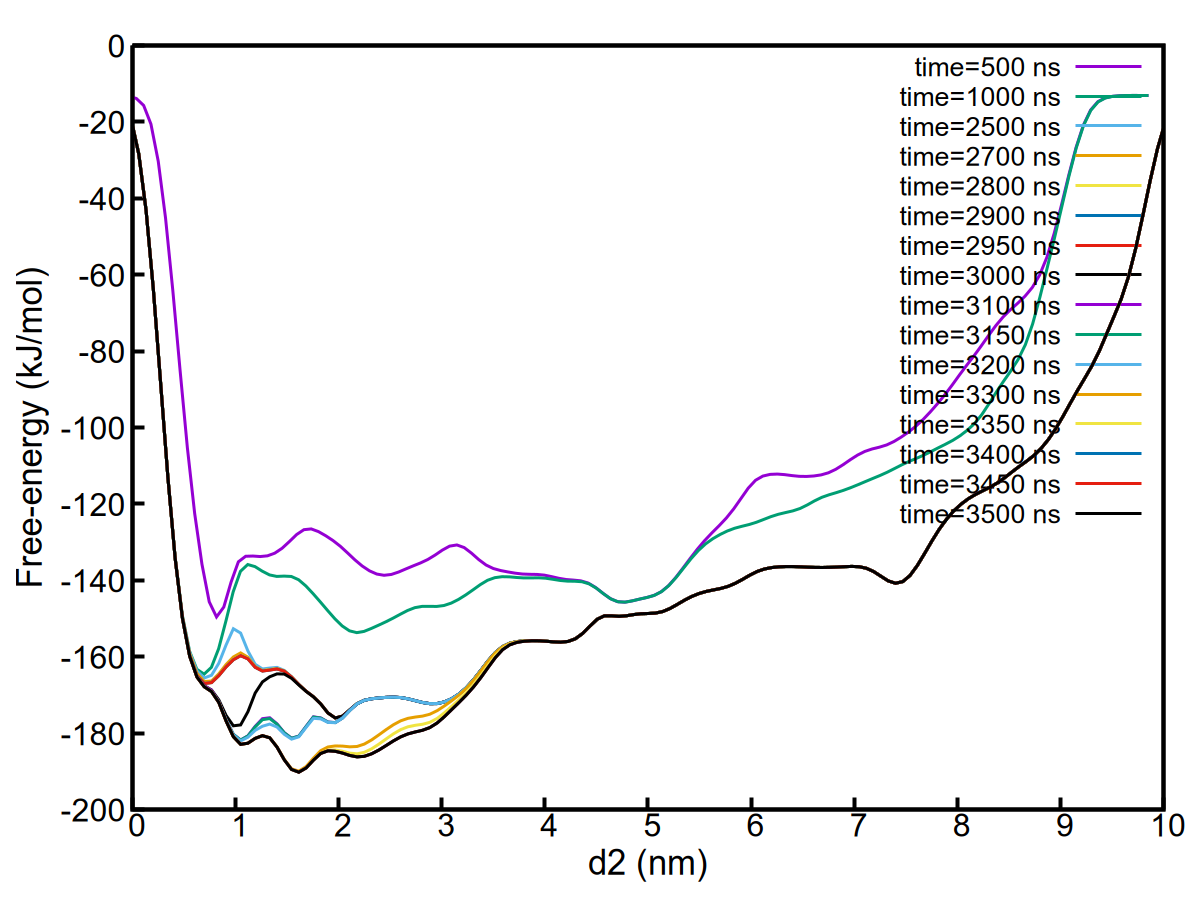

Supplement: Supplementary file 1 [file ci5c02155_si_001.zip › Supporting_infomation/SI-fig5-b.png]

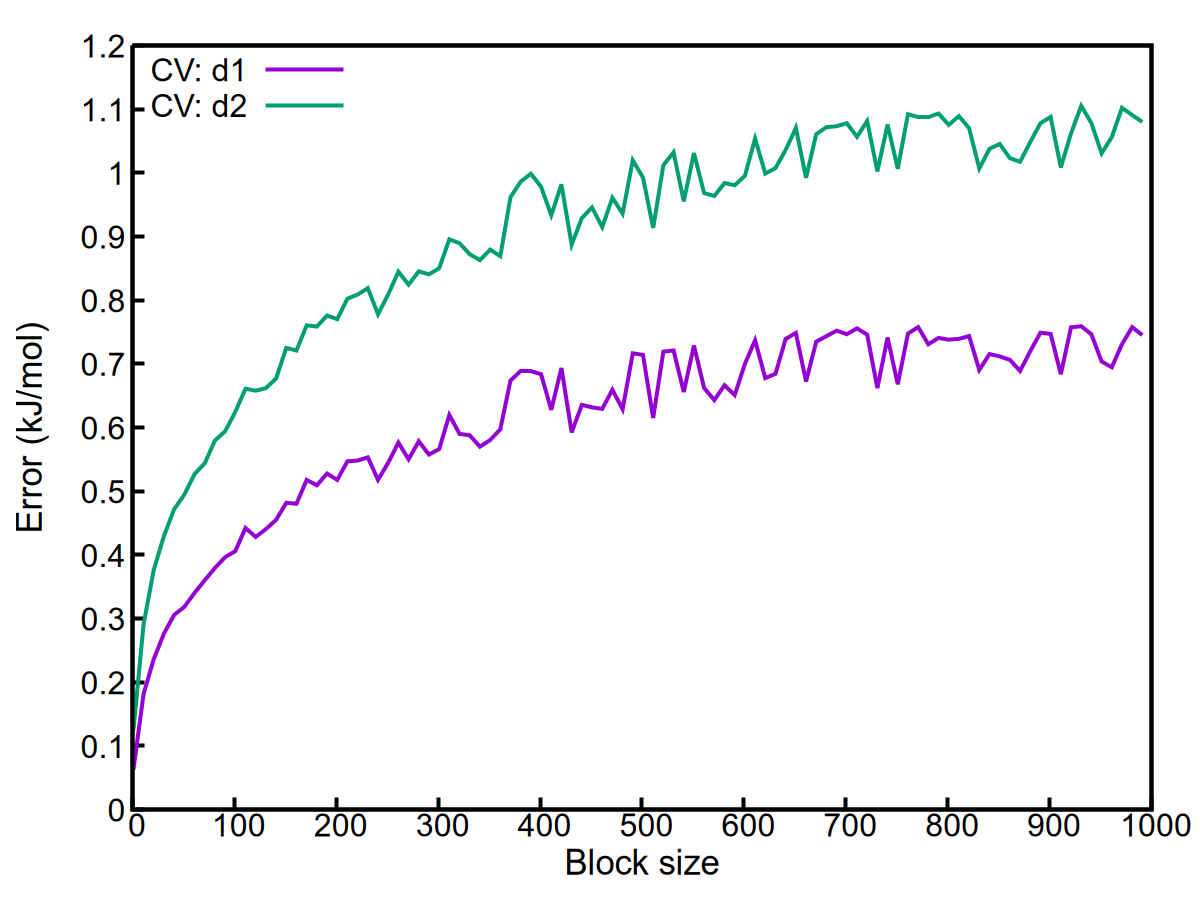

Supplement: Supplementary file 1 [file ci5c02155_si_001.zip › Supporting_infomation/SI-fig6.png]

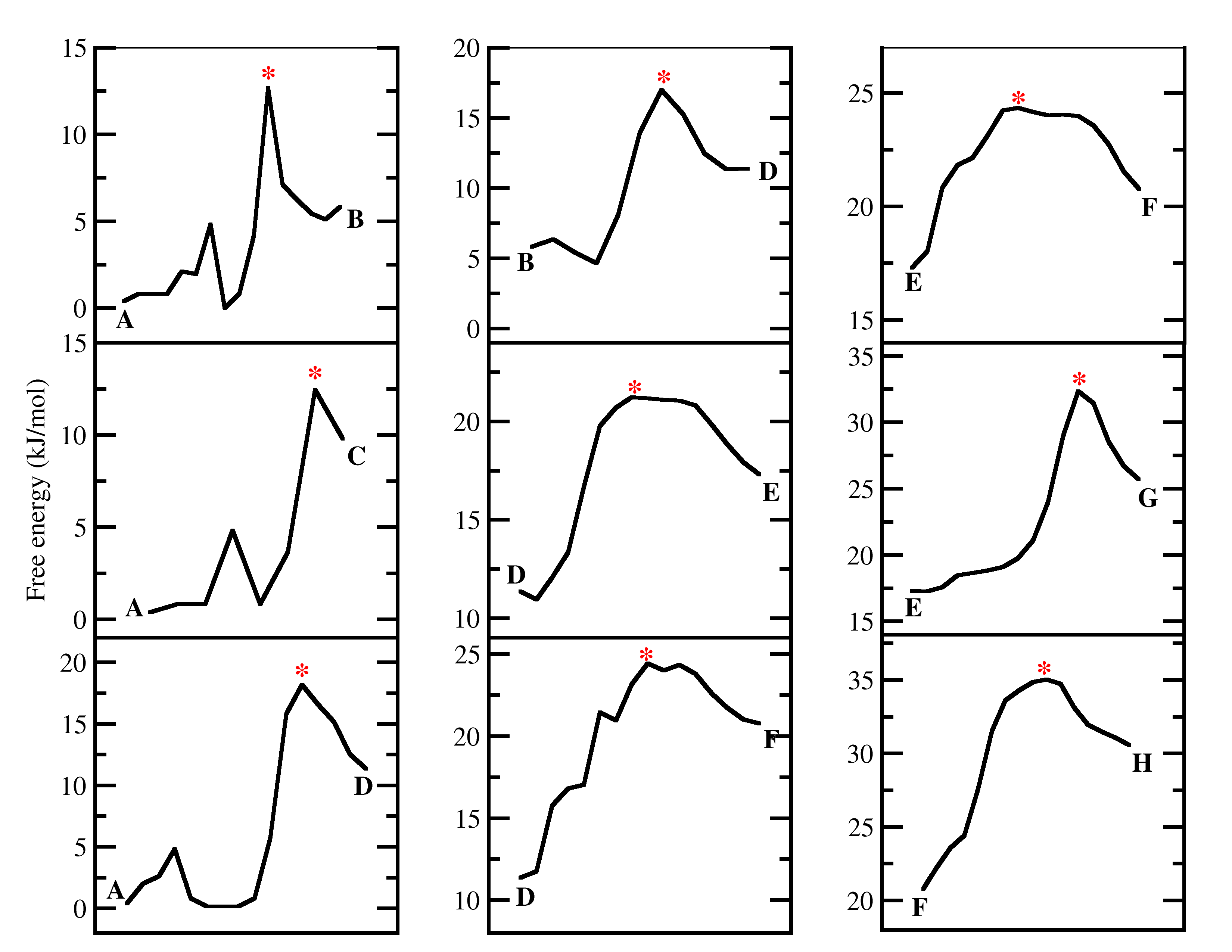

Supplement: Supplementary file 1 [file ci5c02155_si_001.zip › Supporting_infomation/SI-fig7.png]

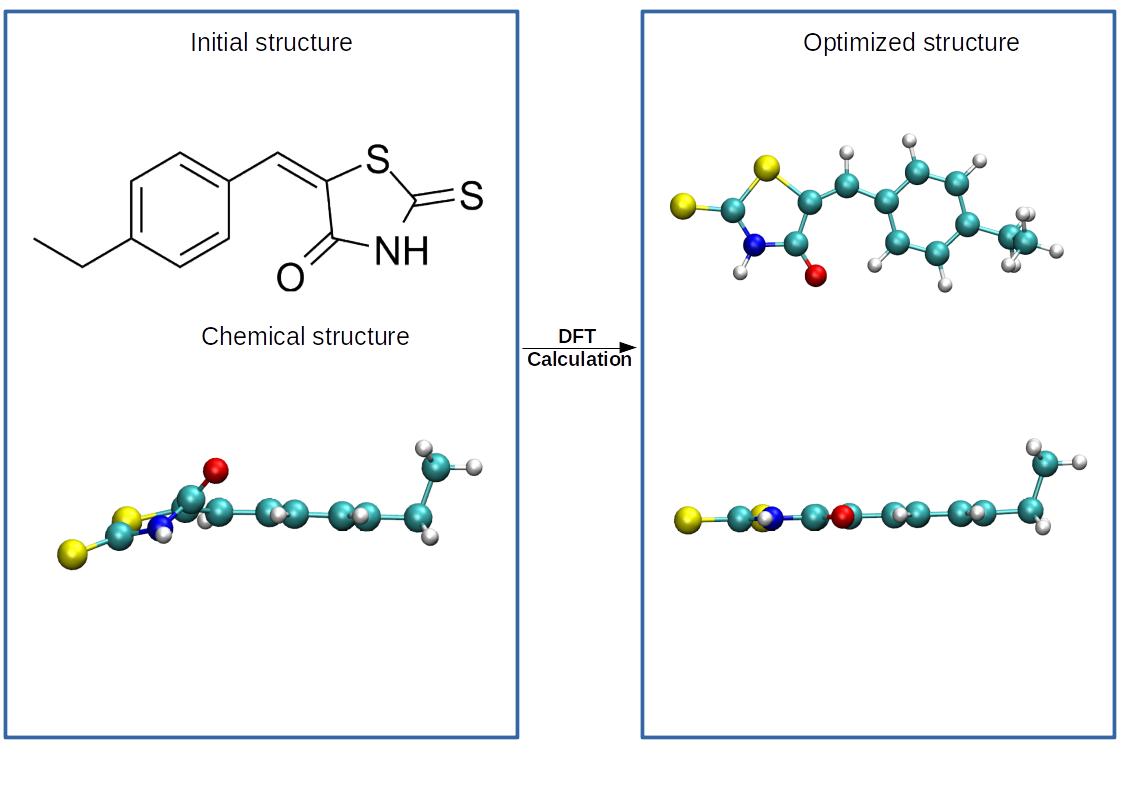

Supplement: Supplementary file 1 [file ci5c02155_si_001.zip › Supporting_infomation/SI-fig8.png]

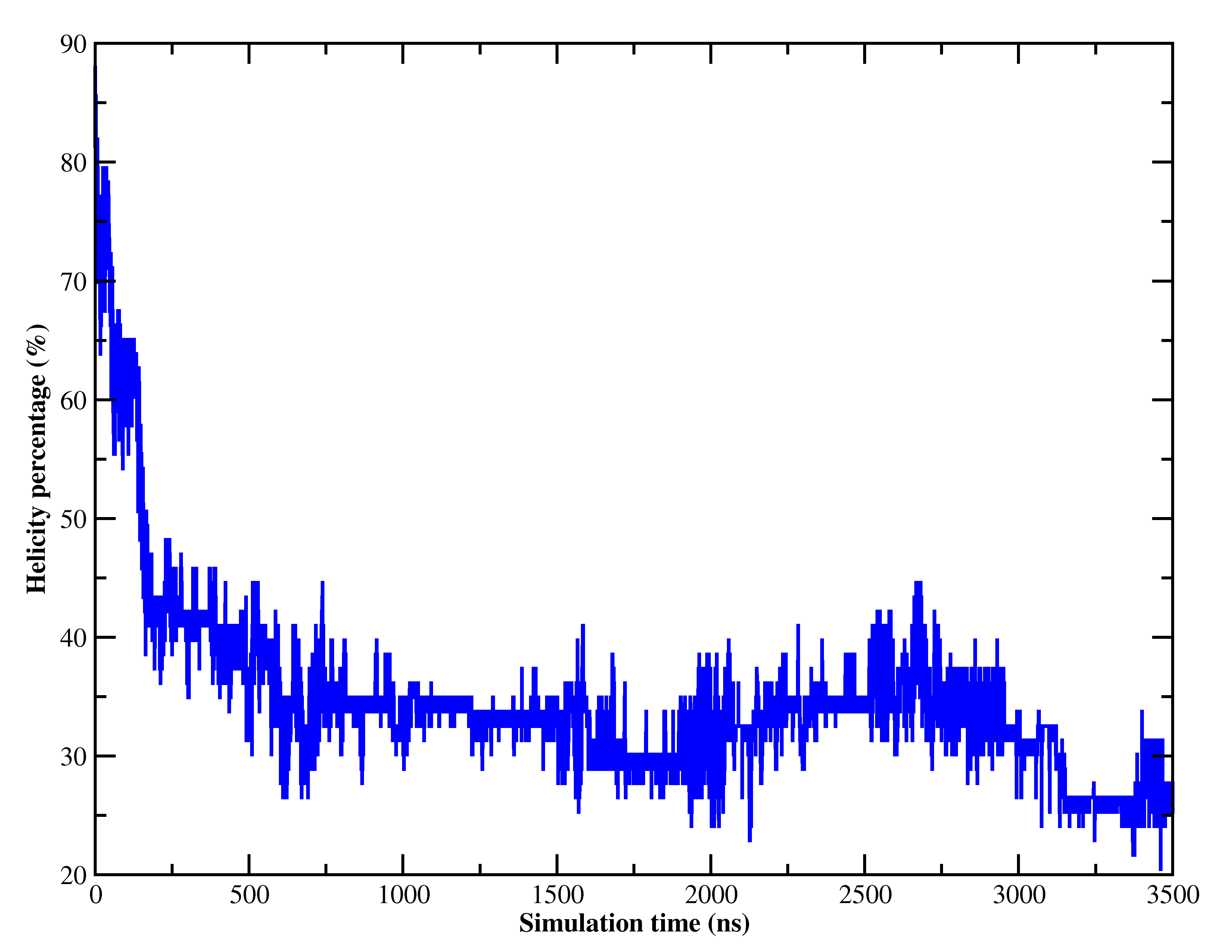

Supplement: Supplementary file 1 [file ci5c02155_si_001.zip › Supporting_infomation/SI-helicity.png]
